# Passive Leg Raising Correlates with Future Exercise Capacity after Coronary Revascularization

**DOI:** 10.1371/journal.pone.0137846

**Published:** 2015-09-11

**Authors:** Shu-Chun Huang, May-Kuen Wong, Pyng-Jing Lin, Feng-Chun Tsai, Ming-Shien Wen, Chi-Tai Kuo, Chih-Chin Hsu, Jong-Shyan Wang

**Affiliations:** 1 Department of Physical Medicine and Rehabilitation, Chang Gung Memorial Hospital, Linkuo, Taoyuan, Taiwan; 2 Division of Thoracic and Cardiovascular Surgery, Gung Memorial Hospital, Linkuo, Taoyuan, Taiwan; 3 Second Section of Cardiology, Department of Medicine, Chang Gung Memorial Hospital, Linkuo, Taoyuan, Taiwan; 4 First Cardiovascular Division, Department of Cardiology, Chang Gung Memorial Hospital, Linkuo, Taoyuan, Taiwan; 5 Department of Physical Medicine and Rehabilitation, Chang Gung Memorial Hospital, Keelung, Taiwan; 6 Healthy Aging Research Center, Graduate Institute of Rehabilitation Science, Medical College, Chang Gung University, Taoyuan, Taiwan; Boston University, UNITED STATES

## Abstract

Hemodynamic properties affected by the passive leg raise test (PLRT) reflect cardiac pumping efficiency. In the present study, we aimed to further explore whether PLRT predicts exercise intolerance/capacity following coronary revascularization. Following coronary bypass/percutaneous coronary intervention, 120 inpatients underwent a PLRT and a cardiopulmonary exercise test (CPET) 2–12 days during post-surgery hospitalization and 3–5 weeks after hospital discharge. The PLRT included head-up, leg raise, and supine rest postures. The end point of the first CPET during admission was the supra-ventilatory anaerobic threshold, whereas that during the second CPET in the outpatient stage was maximal performance. Bio-reactance-based non-invasive cardiac output monitoring was employed during PLRT to measure real-time stroke volume and cardiac output. A correlation matrix showed that stroke volume during leg raise (SV_LR_) during the first PLRT was positively correlated (R = 0.653) with the anaerobic threshold during the first CPET. When exercise intolerance was defined as an anaerobic threshold < 3 metabolic equivalents, SV_LR_ / body weight had an area under curve value of 0.822, with sensitivity of 0.954, specificity of 0.593, and cut-off value of 1504·10^-3^mL/kg (positive predictive value 0.72; negative predictive value 0.92). Additionally, cardiac output during leg raise (CO_LR_) during the first PLRT was related to peak oxygen consumption during the second CPET (R = 0.678). When poor aerobic fitness was defined as peak oxygen consumption < 5 metabolic equivalents, CO_LR_ / body weight had an area under curve value of 0.814, with sensitivity of 0.781, specificity of 0.773, and a cut-off value of 68.3 mL/min/kg (positive predictive value 0.83; negative predictive value 0.71). Therefore, we conclude that PLRT during hospitalization has a good screening and predictive power for exercise intolerance/capacity in inpatients and early outpatients following coronary revascularization, which has clinical significance.

## Introduction

Accumulated evidence supports that early exercise training can reduce postoperative complications and shorten the hospital stay after coronary revascularization surgery in cardiac patients [1,2]. The necessity of aggressive rehabilitation is especially important in patients with poor functional outcome. Nonetheless, there is a lack of a convenient and objective strategy for predicting exercise intolerance/capacity and subsequently deciding the timing and intensity of an initial exercise program following coronary revascularization.

Passive leg raise (PLR) involves raising the legs of a patient without the patient’s active participation; this causes gravity to aid venous return from the lower limbs, thus increasing circulatory volume available to the heart [3]. In heathly subjects, a study using radionuclide method demonstrated that the volume transferred during 45-degree leg raise from the horizontal plane was about 150 ml [4]. Real-time detection of the effect of this maneuver on hemodynamic characteristics has been reported to be useful in making the decision on whether additional fluid is beneficial in critically ill patients [3,6–8]. An echocardiographic study conducted in eu-volemic coronary artery disease patients, proved that PLR increases preload and, consequently, cardiac performance [5]. How stroke volume responds to PLR depends on the Frank-Starling mechanism, including cardiac contractility and preload [3]. Bertolissi et al. further confirmed that cardiac index is unchanged during PLR in those with reduced right ventricular ejection fraction [9]. Moreover, PLR may also identify impairment of diastolic functional reserve during exercise in patients with abnormal myocardial relaxation [10]. However, few studies have addressed the relationships between exercise intolerance and PLR-induced hemodynamic changes in patients with cardiovascular disorders.

The PLR test (PLRT) primarily and passively uses gravity to stimulate the heart, whereas a cardiopulmonary exercise test (CPET) simultaneously exerts active muscle pumping and autonomic cardiovascular responses to the heart [5,11]. Since the Frank-Starling mechanism is disturbed in a diseased heart, we hypothesized that decreased PLR-related cardiac hemodynamic changes would be closely linked to functional aerobic impairment in patients with cardiovascular disorders. Hence, in the present study, we aimed to evaluate whether PLR-induced hemodynamic changes reflect functional aerobic capacity, thus establishing an effective, predictive index for exercise intolerance following coronary revascularization in cardiac patients.

## Methods

### Participants and study protocol

This was a longitudinal cohort study involving 120 hospitalized patients admitted for percutaneous coronary intervention or coronary artery bypass grafting in Chang Gung Memorial hospital. Patients with contraindications to exercise testing were excluded, including life-threatening cardiac arrhythmias, decompensated heart failure, uncontrolled hypertension, advanced atrioventricular block, acute myocarditis and/or pericarditis, symptomatic aortic stenosis, severe hypertrophic obstructive cardiomyopathy, acute systemic illness, and intra-cardiac thrombus. Two to twelve days after coronary revascularization surgery, while still hospitalized, participants underwent a first PLRT using a bio-reactance-based noninvasive cardiac output monitor (NICOM), immediately followed by a submaximal CPET. This first CPET was terminated upon reaching ventilatory anaerobic threshold (VAT). Three to five weeks after leaving the hospital, subjects underwent a second PLR, followed by a maximal CPET. Between the two tests, patients received optimal medical treatment and underwent conventional inpatient and outpatient cardiac rehabilitation, including conditioning exercise, respiratory training, education, and cardiovascular risk factor control [12]. The investigation was performed according to the Helsinki declaration and was approved by the Institutional Review Board of Chang Gung Memorial Hospital, Taiwan. All relevant ethical safeguards have been met in relation to patient or subject protection. After we meticulously explained the purposes of this study to the subjects, all subjects signed informed consent forms before participating.

### Passive leg raise test

**Fig 1:**
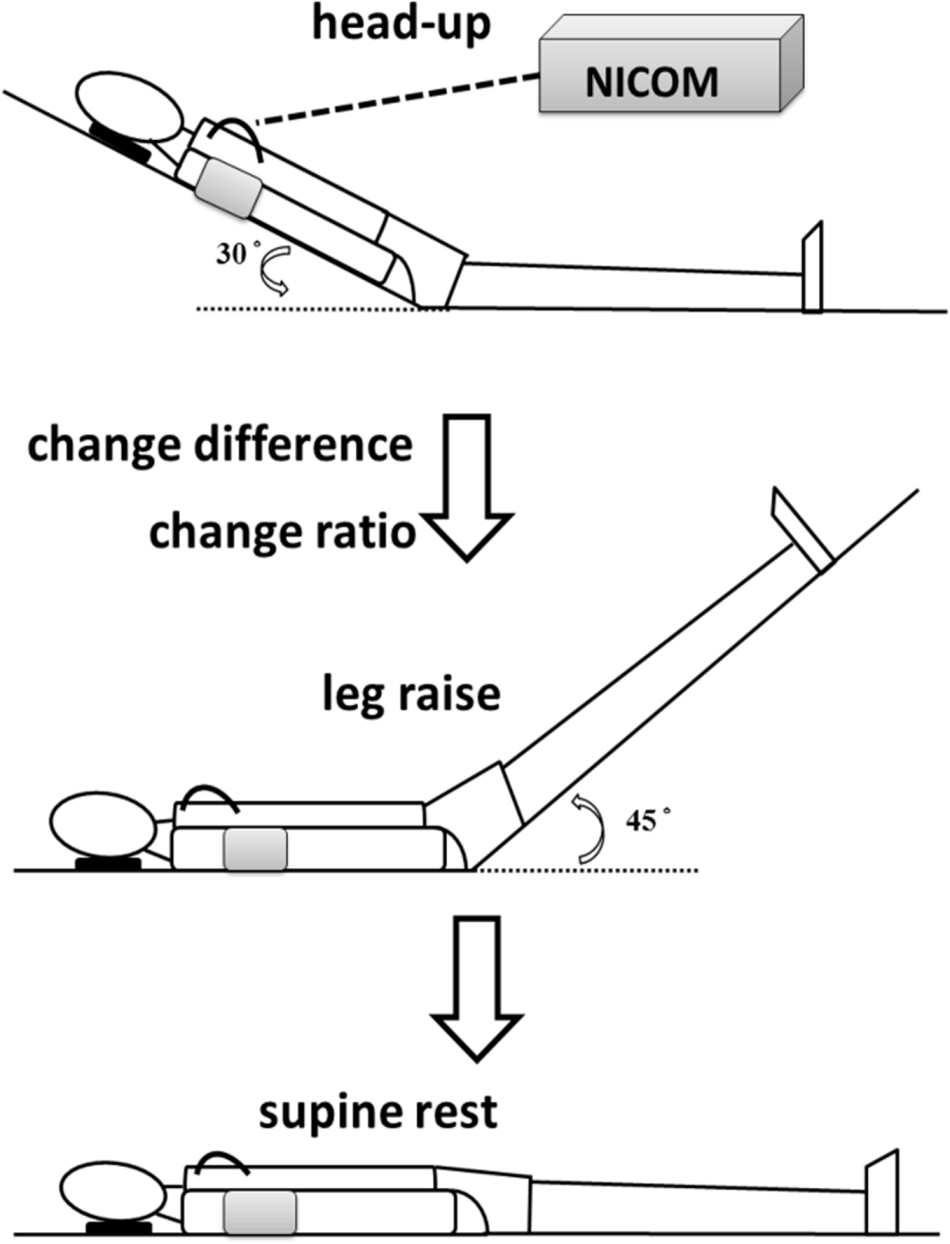
Illustration demonstrating performance of the passive leg raise test.

The PLRT involves the patient assuming three consecutive postures at five-minute intervals: head-up (HU), leg raise (LR), and supine rest (SR) (Fig 1). During HU, the participant lies down on a wedge-shaped backrest angled 30° to the horizontal plane for 5 minutes. Next, bilateral lower extremities of the subject are passively elevated to 45° (LR) for 5 minutes, and then returned to SR for another 5 minutes. During each interval, hemodynamic responses were assessed every minute, including heart rate (HR), stroke volume (SV), cardiac output (CO), total peripheral resistance (TPR), thoracic fluid content, and systolic and diastolic blood pressures. LR values are those values coincident with the largest SV, which was usually between the second and fourth minutes. LR5 values denote the value at the fifth minute of LR. HU and SR values are the average among values during 3^rd^, 4^th^and 5^th^ minutes. Change difference (CD) and change ratio (CR) of the hemodynamic parameters between HU and LR were calculated as follows: CD = LR–HU, and CR = CD/HU. During PLRT, NICOM was employed to evaluate real-time cardiac hemodynamics.

### Cardiac hemodynamic measurements

Cardiac hemodynamics were measured by NICOM^Ra^ (Cheetah Medical, Wilmington, Delaware, USA). The validity and reliability of CO measurement at rest in patients with heart failure were previously demonstrated, and its use has been reported in several studies [13–16]. The regression of CO measured from the NICOM compared to that measured from the thermos-dilution method for 27 cardiac patients showed a good correlation (R = 0.9) [13]. Four dual-surface electrodes were placed on the anterior chest to establish electrical contact with the body. The phase shifts (dΦ) derived from the contraction of the left ventricle followed by blood flow into the thoracic aorta were recorded. The greater the cardiac SV, the more significant these phase shifts become. Therefore, this device measures SV (mL/beat) and CO (L/min) in a non-invasive, continuous, and real-time manner. SV is estimated by the product of C VET dΦ/dtmax, where C is a constant of proportionality and VET denotes the ventricular ejection time determined from the NICOM and electrocardiogram signals. Other hemodynamic variables were calculated using the following equation: CO = SV × HR, TPR = mean arterial pressure (MAP)/CO, SV compliance (SVCom) = SV/pulse pressure [17].SV index (SVI) = SV/body surface area (BSA), Cardiac index (CI) = CO/BSA, BSA(m^2^) = 0.20247 ×height(m)^0.725^× weight(kg)^0.425^ [18].TPR index (TPRI) = MAP/CI, and SVI compliance (SVICom) = SVI/pulse pressure [17].

### Cardiopulmonary exercise testing

Subjects underwent the CPET graded exercise test in an upright position on a calibrated bicycle ergometer 2–4 hours after a light meal (Ergoselect 150P, Germany) to assess aerobic fitness. The test began with 2 min of rest and 1 min of warm-up at 10 Watt followed by a 10 Watt ramp increase in the work rate every minute until exhaustion. Minute ventilation (V_E_), oxygen consumption (VO_2_), and carbonic dioxide production (VCO_2_) were measured, breath by breath, using a computer-based system (MasterScreen CPX, Cardinal-health Germany). HR was determined from the R-R interval on a 12-lead electrocardiogram, arterial pressure was measured using an automatic blood pressure system (Tango, SunTech Medical, UK), and arterial O_2_ saturation was monitored by finger pulse-oximetry (model 9500, Nonin Onyx, Plymouth, Minnesota).VO_2peak_ was defined by the following criteria: (i) VO_2_ increased by less than 2 mL/kg/min over at least 2 min, (ii) HR exceeded 85% of the predicted maximum, (iii) the respiratory exchange ratio exceeded 1.15, or (iv) some other symptom/sign limitations [19]. VAT was determined by two experienced, independent reviewers using the V-slope method and verified based on ventilatory criteria as follows: (i) the V_E_/VO_2_ ratio increased without a corresponding increase in the V_E_/VCO_2_ ratio, (ii) P_ET_O_2_ increased without a decrease in the P_ET_CO_2_, or (iii) a departure from linearity for V_E_ [20]. Exercise intolerance was defined as VO_2VAT_ < 3 metabolic equivalents (METs) or peak VO_2_ < 5METs based on New York Heart Association criteria [21–22].

### Validity and reliability

Validity of bio-reactance-based NICOM (non-invasive cardiac output monitor) was verified against echocardiography by biplane Simpson method under apical four-chamber view and long-axis view. 11 male patients (age 56 ± 13 years) following coronary revascularization were enrolled. During PLRT, SV and CO were simultaneously obtained by these two methods at HU, LR and SR. Test-retest reliability of PLRT by NICOM was performed in separate days in six patients with coronary artery disease (CAD) and three healthy participants.

### Statistics

Data are expressed as mean ± standard error of mean and were analyzed using SPSS 18.0 software. A correlation matrix was used to compare all the hemodynamic variables obtained in the first PLRT against the submaximal CPET and those obtained during the first PLRT against the maximal CPET. The most relevant hemodynamic values during PLRT were chosen to calculate the area under curve. Receiver operating characteristic (ROC) curve analysis was employed to determine hemodynamic variables at PLRT in diagnosing VO_2VAT_ < 3 METs and VO_2peak_ < 5METs. A paired *t*-test was used to compare the first and second PLRT. All hemodynamic variables from the same PLRT at LR, LR5, and SR were compared to those at HU with a paired *t*-test. The criterion for significance was P < .05. Bland-Altman plot was used in assessing agreement between values obtained by NICOM and echocardiography in validity testing. The single-measure intra-class correlation was employed in test-retest reliability of PLRT.

## Results

Demographic data of the study cohort is shown in Table 1. A total of 120 patients participated in the investigation and underwent the first PLRT and submaximal CPET. Of these, 12 patients dropped out due to complications, their own unwillingness to undergo the test, or loss to follow-up; thus, 108 patients underwent the second PLRT and maximal CPET.

**Table 1:** Demographic data.

**Gender**	N (M/F)	120 (98/22)
**Age**	year	55.5±1.4
**Height**	cm	161.8±2.1
**Weight**	Kg	65.0±1.1
**BMI**	Kg/m^2^	25.6±1.1
**Surgery**		
**coronary bypass**	N	86
**percutaneous coronary intervention**	N	34
**Comorbidity**		
**diabetes**	N (%)	86(72%)
**hypertension**	N (%)	55(46%)
**Echocardiography**		
**LVEF,%**	%	53.7±1.2
**LVEDD**		50.4±0.8
**LVESD**		34.7±0.8
**Medications**		
**β-blockers**	N (%)	110(92%)
**ACE/ARB**	N (%)	79(66%)
**CCB**	N (%)	32(27%)
**Diuretics**	N (%)	67(56%)

Data is expressed as mean ± standard error of mean

**Fig 2:**
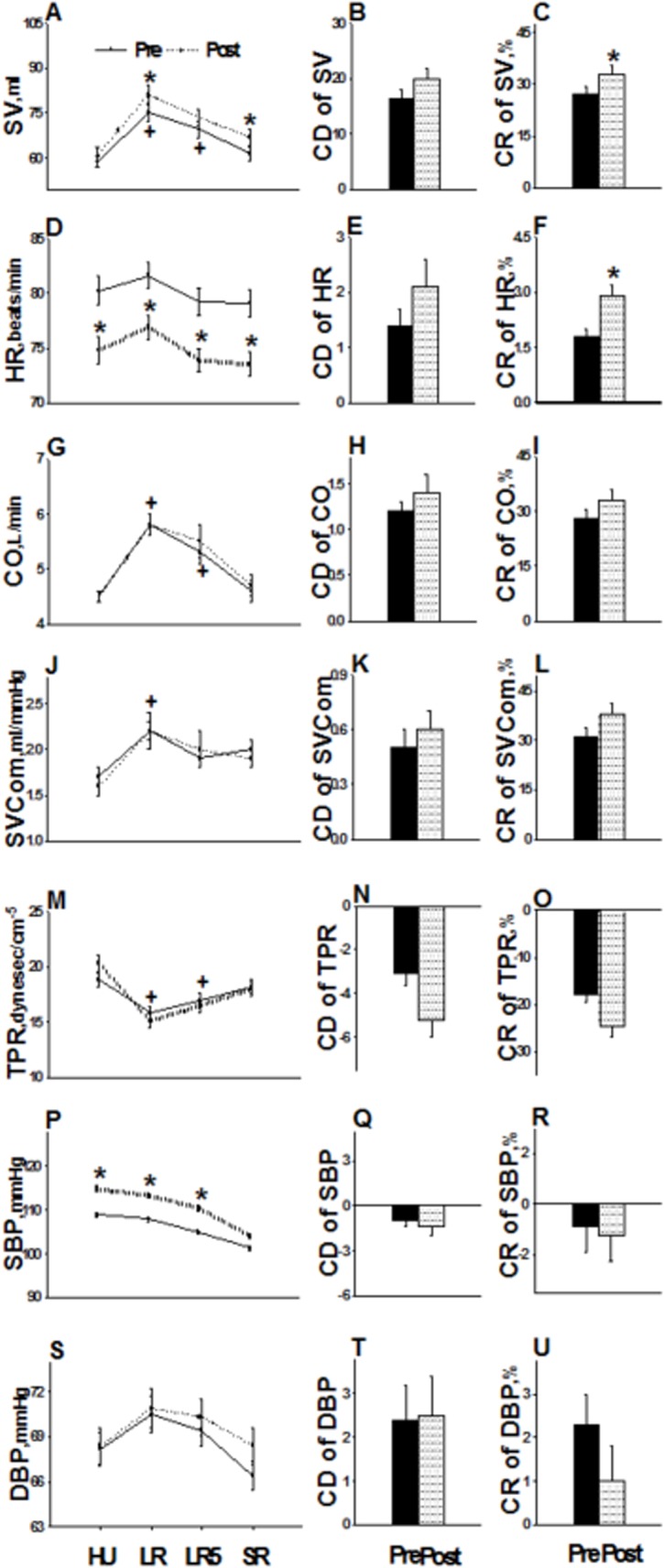
Hemodynamic responses to passive leg raising during hospitalization (pre) and following discharge (post). **HU**: head-up (HU values are the average among 3^rd^~5^th^ minutes); **LR**: leg raise (LR values are those values coincident with the largest SV); **LR5**: leg raise at the 5th minute; **SR**: supine rest (SR values are the average among 3^rd^~5^th^ minutes). **CD**: change difference from HU to LR; **CR**: change ratio from HU to LR = CD/HU). (**A**) SV(stroke volume) at HU, LR, LR5 and SR; (**B**) CD of SV; (**C**) CR of SV. Likewise, (**D**)~(**F**) HR: heart rate; (**G**)~(**I**) CO: cardiac output; (**J**)~(**L**) SVCom: stroke volume compliance; (**M**)~(**O**) TPR: total peripheral resistance; (**P**)~(**R**) SBP: systolic blood pressure; (**S**)~(**U**) DBP: diastolic blood pressure.

Fig 2 illustrates the hemodynamic response to PLR. From HU to LR, primary changes included increased SV, CO, and SVCom and decreased TPR. The changes in HR, systolic and diastolic BP from HU to LR were not significant. The largest change in hemodynamic variables from HU to LR usually occurred between the second and fourth minutes during LR. When comparing the hemodynamic response to PLR in the first(inpatient) and second(outpatient) tests, there were significant increases in SV_LR_, SV_SR_, SV_CR_, and HR_CR_ as well as decreases in HR_HU_, HR_LR_, HR_LR5_, and HR_SR_.

**Fig 3:**
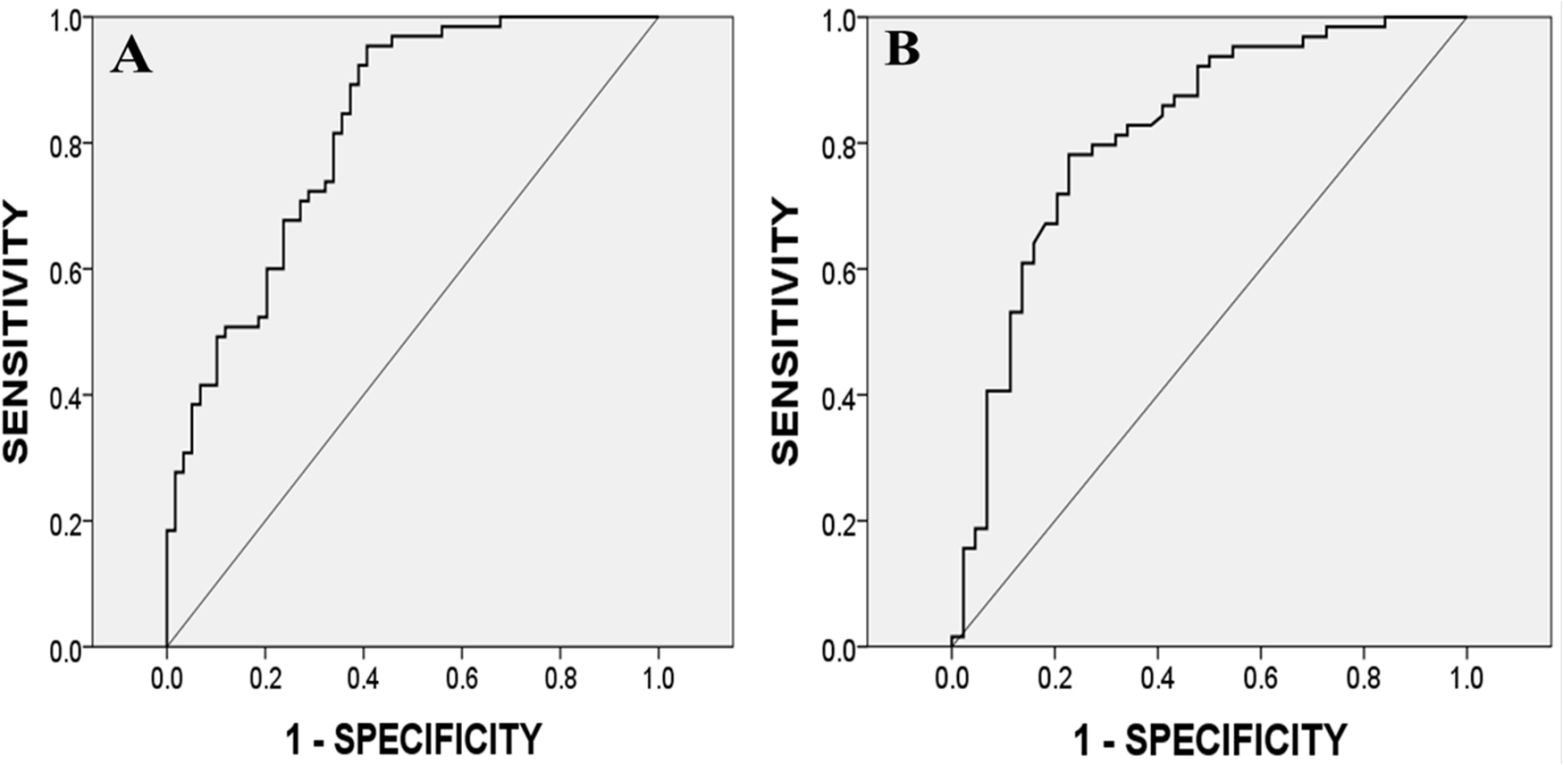
Receiver operating characteristic analysis for exercise intolerance by passive leg raise tests following coronary revascularization. **(A)** Receiver operating characteristic curve of the SV_LR_/body weight during 1^st^ PLRT in predicting poor aerobic fitness at 1st CPET with VAT<3METs. A cut-off value of 1504·10^−3^ ml/kg resulted in sensitivity of 0.954, specificity of 0.593 and the area under the curve of 0.822 for the measurement. **(B)** ROC curve of the CO_LR_/body weight during 1^st^ PLRT in predicting poor endurance at 2nd CPET with peak VO_2_<5METs. A cut-off value of 68.3 mL/min/kg resulted in sensitivity of 0.781, specificity of 0.773, and the AUC of 0.814 for the measurement.

Some hemodynamic variables during the first PLRT were associated with VO_2VAT_ during the corresponding submaximal CPET (Table 2). Among them, SV has the highest correlation coefficients followed by SVCom and CO. The correlation coefficients of LR were the highest when compared to those at HU, LR5, SR, CD, and CR. Stroke volume at LR (SV_LR_) and the first VO_2VAT_ showed the highest correlation coefficient value (R = 0.653). Lesser degree of correlation was noted between the first VO_2VAT_ and SVI, SVIcom, CI, thoracic fluid content, TPR, TPRI, HR, systolic blood pressure, diastolic blood pressure, and MAP at HU, LR, LR5, SR, CR, and CD (Table 2). Additionally, resting left ventricular ejection fraction by echocardiography correlated poorly with workload_VAT_ (R = 0.15). The feasibility of using PLRT to screen those with exercise intolerance was performed by ROC analysis. VO_2VAT_ < 3 METs was defined as exercise intolerance (positive). Table 3 shows that SV_LR_ had the greatest AUC (0.84). The corresponding ROC curve regarding SV_LR_/body weight (BW) versus exercise intolerance is shown in Fig 3A. The AUC is 0.82 and a cut-off value of 1504·10^−3^ ml/kg resulted in sensitivity of 0.954 and specificity of 0.593. The positive predictive value was 0.72, and the negative predictive value was 0.92 in the present cohort.

**Table 2:** Correlation matrix of multiple variables at 1^st^ PLRT vs. VO_2VAT_ at 1^st^ submaximal CPET.

	HU	LR	LR5	SR	CD	CR
**SV**	0.588**	0.653**	0.632**	0.591**	0.505**	0.271**
**SVCom**	0.489**	0.569**	0.519**	0.379**	0.499**	0.242**
**CO**	0.392**	0.541**	0.497**	0.483**	0.460**	0.310**
**TFC**	-0.216*	-0.192*	-0.190*	-0.203*	0.203**	0.206*
**TPR**	-0.331**	-0.471**	-0.455**	-0.430**	-0.196**	-0.352**
**HR**	-0.430**	-0.420**	-0.416**	-0.416**	0.225**	0.163
**SBP**	-0.092	-0.065	0.016	-0.084	-0.065	-0.081
**DBP**	0.016	0.100	0.026	0.003	0.163	0.156
**MAP**	-0.012	-0.092	0.009	-0.054	0.224**	0.152
**SVI**	0.479**	0.572**	0.548**	0.493**	0.462**	0.271**
**SVICom**	0.394**	0.487**	0.417**	0.303**	0.454**	0.242**
**CI**	0.179	0.399**	0.363**	0.316**	0.411**	0.310**
**TPRI**	-0.164	-0.362**	-0.348**	-0.292**	-0.251**	-0.352**

*p < 0.05

** *p* < 0.01

VAT: ventilatory anaerobic threshold; CD: change difference from HU to LR; CR: change ratio from HU to LR = CD/HU; HU: head-up; LR: leg raise; LR5: leg raise at the 5th minute; SR: supine rest; CO: cardiac output; CI: cardiac index; DBP: diastolic blood pressure; HR: heart rate; MAP: mean arterial pressure; SBP: systolic blood pressure; SV: stroke volume; SVCom: stroke volume compliance; SVIcom: stroke volume index compliance; TFC: thoracic fluid content; TPR: total peripheral resistance; TPRI: total peripheral resistance index

**Table 3:** Area under curve of hemodynamic variables during 1^st^ PLRT and VO_2VAT_ at 1st CPET.

	HU	LR	LR5	SR	CD	CR
**SV**	0.769	0.840	0.801	0.762	0.734	0.637
**SVCom**	0.699	0.731	0.705	0.726	0.716	0.633
**CO**	0.657	0.746	0.729	0.732	0.707	0.66
**TFC**	0.461	0.469	0.467	0.451	0.570	0.566
**TPR**	0.391	0.295	0.311	0.343	0.387	0.343
**SVI**	0.612	0.644	0.641	0.610	0.624	0.637
**SVICom**	0.605	0.625	0.575	0.591	0.616	0.633
**CI**	0.553	0.642	0.622	0.608	0.614	0.660
**TPRI**	0.401	0.359	0.377	0.374	0.402	0.343

VAT: ventilatory anaerobic threshold; CD: change difference from HU to LR; CR: change ratio from HU to LR = CD/HU; HU: head-up; LR: leg raise; LR5: leg raise at the 5th minute; SR: supine rest; CO: cardiac output; CI: cardiac index; SV: stroke volume; SVCom: stroke volume compliance; SVIcom: stroke volume index compliance; TFC: thoracic fluid content; TPR: total peripheral resistance; TPRI: total peripheral resistance index

**Fig 4:**
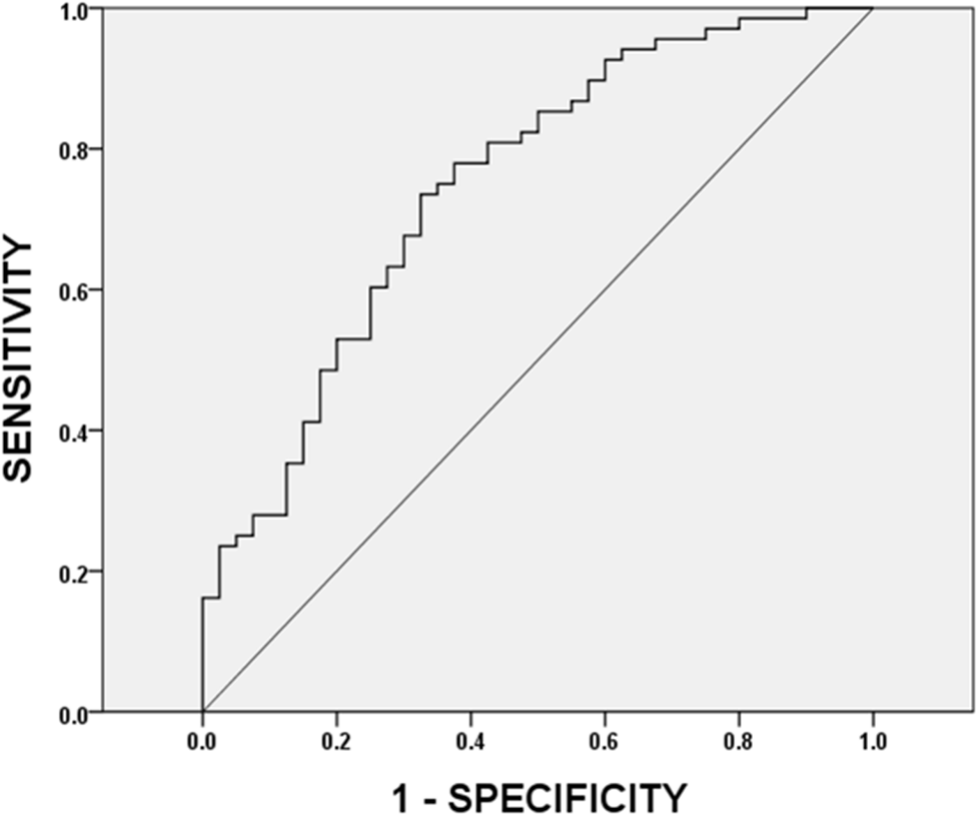
Receiver operating characteristic curve analysis between CI_LR_ during 1st PLR vs. exercise intolerance during 2nd CPET. ROC curve of the CI_LR_ during 1st PLRT in predicting poor aerobic fitness at 2nd CPET with VAT < 5METs is shown. AUC of the measurement is 0.749. A cut-off value of 3.9 liter/min/meter^2^ resulted in sensitivity of 0.779 and specificity of 0.625. The positive and negative predictive values are 0.78 and 0.61 respectively in the study cohort. CI_LR_: cardiac index during leg raise

Hemodynamics during the first PLRT was as well associated with peak VO_2_ in the second, maximal CPET (Table 4). Among them, CO has the greatest correlation, followed by SVCom and SV. CO at LR (CO_LR)_ had the highest value of correlation coefficient (R = 0.678) versus VO_2peak_. There were lesser degree of correlation between CI, SVI, SVIcom, thoracic fluid content, TPR, and TPRI at HU, LR, LR5, SR, CR, and CD compared to peak VO_2_ during the second CPET (Table 4). The usefulness of PLRT to predict exercise intolerance was performed by an area under curve analysis. UsingVO_2peak_ < 5 METs to define exercise intolerance (positive), which corresponds to the New York Heart Association [23] classification of Heart Failure II, III, or IV, Table 5 shows that CO_LR_ had the greatest AUC (0.83). Its corresponding ROC curve concerning CO_LR_/BW and exercise intolerance is shown in Fig 3B. The AUC is 0.81 and a cut-off value of 68.3 ml/min/kg resulted in sensitivity of 0.781 and specificity of 0.773. The positive predictive value was 0.83, and the negative predictive value was 0.71 in the study cohort. Notably, as we know, CI reflects cardiac function. Unexpectedly, however, CI had lower degree of correlation and AUC versus aerobic fitness than SV/BW in the 1^st^ PLR vs. 1^st^ CPET comparison (CI vs. SV/BW: R = 0.399 vs. 0.653; AUC = 0.642 vs. 0.822, Tables 2 and 3), and also CO/BW in the 1^st^ PLR vs. 2^nd^ CPET comparison (CI vs. CO/BW; R = 0.556 vs. 0.678; AUC = 0.749 vs. 0.814, Tables 4 and 5). ROC curve analysis between CI at LR during 1^st^ PLR vs. exercise intolerance during 2^nd^ CPET was demonstrated in Fig 4.

**Table 4:** Correlation matrix of hemodynamic variables at 1^st^ PLRT vs. peak VO_2_ at 2^nd^ maximal CPET

	HU	LR	LR5	SR	CD	CR
**SV**	0.559**	0.526**	0.562**	0.437**	0.403*	0.266
**SVCom**	0.372*	0.603**	0.604**	0.551**	0.240	0.580**
**CO**	0.240	0.678**	0.634**	0.644**	0.469**	0.614**
**TFC**	0.371*	0.252	0.323	-0.13	0.112	0.104
**TPR**	-0.126	0.189	0.202	-0.511**	-0.498**	-0.537**
**SVI**	0.489**	0.534**	0.495**	0.367*	0.266	0.266
**SVICom**	0.364*	0.594**	0.563**	0.495**	0.546**	0.580**
**CI**	0.252	0.556*	0.524**	0.549**	0.521**	0.614**
**TPRI**	-0.446**	-0.277	-0.167	-0.390*	-0.496**	-0.537**

*p < 0.05

** *p* < 0.01

CD: change difference from HU to LR; CR: change ratio from HU to LR = CD/HU; HU: head-up; LR: leg raise; LR5: leg raise at the 5th minute; SR: supine rest; CO: cardiac output; CI: cardiac index; SV: stroke volume; SVCom: stroke volume compliane; SVIcom: stroke volume index compliance; TFC: thoracic fluid content; TPR: total peripheral resistance; TPRI: total peripheral resistance index

**Table 5:** Area under curve of hemodynamic variables during 1^st^ PLRT and peak VO_2_ at 2nd CPET.

	HU	LR	LR5	SR	CD	CR
**SV**	0.783	0.812	0.803	0.779	0.769	0.692
**SVCom**	0.749	0.799	0.803	0.806	0.622	0.779
**CO**	0.759	0.832	0.814	0.772	0.672	0.746
**TFC**	0.686	0.672	0.726	0.462	0.452	0.463
**TPR**	0.426	0.634	0.652	0.324	0.253	0.224
**SVI**	0.699	0.806	0.779	0.732	0.696	0.692
**SVICom**	0.639	0.692	0.719	0.699	0.689	0.779
**CI**	0.672	0.749	0.692	0.716	0.763	0.746
**TPRI**	0.321	0.278	0.388	0.405	0.301	0.224

CD: change difference from HU to LR; CR: change ratio from HU to LR = CD/HU; HU: head-up; LR: leg raise; LR5: leg raise at the 5th minute; SR: supine rest; CO: cardiac output; CI: cardiac index; SV: stroke volume; SVCom: stroke volume compliance; SVIcom: stroke volume index compliance; TFC: thoracic fluid content; TPR: total peripheral resistance; TPRI: total peripheral resistance index

**Fig 5:**
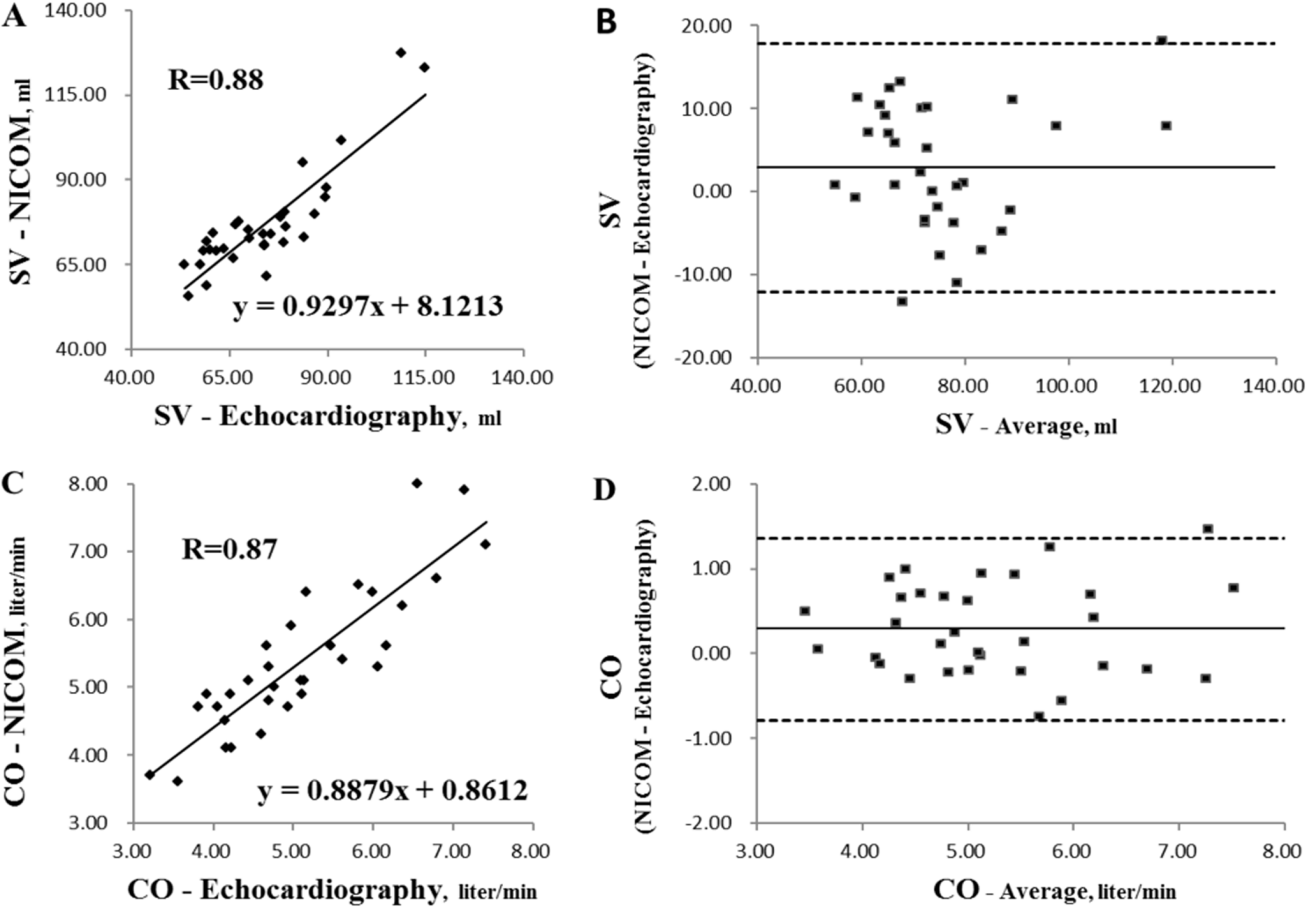
Validity of bio-reactance-based NICOM (non-invasive cardiac output monitor) was verified against echocardiography by biplane Simpson method under apical four-chamber view and long-axis view. Scatter plot of SV and CO between NICOM and echocardiography were demonstrated in **A** and **C**. Agreement using Bland-Altman plot between SV and CO corresponding to NICOM and echocardiography were shown in **B** and **D**. Graph **B** and **D** plot the respective differences between NICOM and echocardiography (y-axis) against the means of these two methods. The dark solid horizontal lines in each Bland-Altman plot represent average bias whereas the dotted lines stand for average bias ± 1.96 standard deviation (95% upper and lower limit). The average bias and standard deviation are 2.9 and 7.6 ml in **B**; and 0.29 and 0.54 liter/min in **D**.

Validity testing showed that the values of SV and CO obtained by NICOM and echocardiography were highly correlated with the slope being close to 1 (Fig 5A and 5C). Bland-Altman plot demonstrated that the average bias ± 1.96 standard deviation (95% upper and lower limit) of SV and CO were 2.9 ± 14.9 ml and 0.29 ± 1.07 liter/min, respectively (Fig 5B and 5D).

Hemodynamic changes during PLRT were highly reproducible, in a day-to-day fashion (n = 9; three healthy and six cardiac patients; age = 51 ± 5 years; gender = seven men and two women). The single-measure intra-class correlations of SV at HU, LR, LR5, and SR were 0.945, 0.972, 0.941, and 0.957, respectively, whereas those of CO at HU, LR, LR5, and SR were 0.936, 0.967, 0.949, and 0.939, respectively, for test-retest reliability.

## Discussion

This is the first study to clearly demonstrate the relationships between PLR-induced hemodynamic changes and exercise capacity/intolerance in cardiac patients following coronary revascularization. Following the coronary revascularization surgery, bio-reactance-based SV_LR_/BW at PLR is capable of estimating submaximal exercise capacity in the inpatient stage, whereas CO_LR_/BW in the inpatient stage serves as a powerful predictor of peak cardiopulmonary fitness in the outpatient stage. The two PLR-related parameters may be employed to screen exercise intolerance with excellent discrimination and high sensitivity.

PLR is considered to passively stretch the myocardium to augment SV or CO, which may be closely relevant to the SV or CO response to physical activity [5,11]. In the current investigation, the extent of change (CD or CR) in SV, CO, and SVCom from HU to LR revealed modest correlations with the first VO_2VAT_ or second VO_2peak_; however, these correlations were consistently lower than those during LR. Accordingly, the present findings imply that submaximal or maximal exercise capacity was associated more with the absolute level of hemodynamics during LR compared to the extent of hemodynamic changes from HU to LR. Therefore, LR-related hemodynamics may serve as an effective index for predicting aerobic fitness in cardiac patients with coronary revascularization.

A previous investigation demonstrated that cardiac patients with E/e' ratios < 15 at rest, but with increasing E/e' ratios > 15 during leg raise, had lower peak VO_2_ as compared to those with persistent E/e' ratios < 15, suggesting that the cardio-dynamic response to preload reflects exercise capacity [10]. The present study further demonstrated that SV at LR serves as a powerful indicator of submaximal exercise performance despite a poor correlation of resting left ventricular ejection fraction with exercise capacity. To date, determination of the timing of starting aerobic exercise training and its intensity following coronary revascularization relies mostly on the clinical judgment of the medical staff. However, according to the present results, PLR can be a simple method to objectively estimate exercise capacity as a clinical practice reference during post-surgery in-hospital rehabilitation. Aggressive rehabilitation should be emphasized in those with poor functional prognosis to facilitate hospital discharge and improve quality of life. Additionally, LR-related hemodynamics may be employed in risk stratification during exercise training.

Notably, the present study further revealed that CO_LR_/BW during hospitalization predicts exercise capacity/exercise intolerance three to five weeks following coronary revascularization, which proactively enables the individualization of an exercise prescription. Several investigations have demonstrated how hemodynamic status determined the efficacy of exercise training in patients with heart failure. Those with normal CO responses to exercise show frequent improvements with exercise training. Patients with severe hemodynamic dysfunction during exercise usually do not improve with training [24]. Furthermore, the presence of chronotropic incompetence predicted an impaired response to exercise training [25]. These studies all highlighted the major role of cardiac performance during active (exercise) or passive (PLR) stress that determines exercise performance or response following exercise training.

When changing position from HU to LR, gravity initiates a shift in blood volume from the lower body to the thorax, thus increasing blood volume of the thoracic (central venous) compartment. This increases cardiac preload, thereby increasing SV and CO, and deactivating the baro-reflex [26]. In turn, systemic vasculature resistance decreases and SVCom increases. At low exertion levels, increased left ventricular filling pressure and end-diastolic volume are also important determinants of the SV response [11] via the Starling mechanism. Thus, PLR and submaximal exercise share similar mechanisms to augment SV.

### Limitations

The current investigation employed NICOM to measure cardiac hemodynamics. Although its validity and reliability have been established in patients with heart failure [13–16,27], invasive methods or echocardiography remain the gold standard. Nonetheless, bio-reactance-based NICOM in PLRT showed excellent test-retest reliability and fair validity versus echocardiography in the present testing. The current findings anyhow showed that bio-reactance-based NICOM combined with PLR can be a simple method to estimate exercise capacity, which can be more convenient for clinical application. Another possible limitation is that, although those with obvious pulmonary or limb edema were excluded, the response of cardiac hemodynamics during PLR may be partly affected by the fluid status, which was not strictly controlled or assessed. However, the hemodynamic changes during PLRT were highly reproducible on the two successive days. Moreover, cardiovascular medications are impossible to control over the 6-week experimental period due to ethical concerns; hence, this may have interfered with the hemodynamic response during PLR and CPET.

### Conclusion

The current investigation provides new insight into the association of PLR and exercise capacity/intolerance. We conclude that PLR performed during hospitalization has a good screening and predictive power in exercise intolerance/capacity following coronary revascularization surgery in the inpatients and 3–5 weeks after hospital discharge.
