# Correction: Passive Leg Raising Correlates with Future Exercise Capacity after Coronary Revascularization

**DOI:** 10.1371/journal.pone.0157205

**Published:** 2016-06-03

**Authors:** Shu-Chun Huang, May-Kuen Wong, Pyng-Jing Lin, Feng-Chun Tsai, Ming-Shien Wen, Chi-Tai Kuo, Chih-Chin Hsu, Jong-Shyan Wang

There are errors in the Funding section. The correct funding information is as follows: This work was supported by Chang Gung Medical Research Program CMRPG 3C1441: HSC; CMRPG 391201–3: HSC; CMRPD 1A0302: WJS; CMRPD 190173: WJS. The funders had no role in study design, data collection and analysis, decision to publish, or preparation of the manuscript.
